# Effect of nutritional status on occurrence of pneumonia after traumatic cervical spinal cord injury

**DOI:** 10.1038/s41598-024-64121-5

**Published:** 2024-06-10

**Authors:** Tetsuo Hayashi, Yuichi Fujiwara, Momo Irie, Muneaki Masuda, Hiroaki Sakai, Hiromitsu Kobayashi, Osamu Kawano, Takeshi Maeda

**Affiliations:** 1 Department of Rehabilitation Medicine, Japan Organization of Occupational Health and Safety, Spinal Injuries Center, Fukuoka, Japan; 2 Department of Nursing, Japan Organization of Occupational Health and Safety, Spinal Injuries Center, Fukuoka, Japan; 3 Department of Orthopedic Surgery, Japan Organization of Occupational Health and Safety, Spinal Injuries Center, Fukuoka, Japan; 4 Department of Assistive Technology and Design Department, Japan Organization of Occupational Health and Safety, Spinal Injuries Center, Fukuoka, Japan; 5 Department of Rehabilitation Medicine, Fukushima Medical University, Fukushima, Japan

**Keywords:** Nutrition, Pneumonia, Spinal cord injury, Respiratory dysfunction, Dysphagia, Immune, Neurological disorders, Respiratory signs and symptoms, Malnutrition

## Abstract

Pneumonia after cervical spinal cord injury (CSCI) is a common and serious complication; however, its nutrition-related etiology has not yet been elucidated. This study aimed to elucidate the effects of nutritional factors on pneumonia after CSCI. Patients with acute traumatic CSCI who were admitted within 3 days after injury and followed up for at least 3 months were retrospectively examined. Occurrence of pneumonia, nutritional status, severity of dysphagia, vital capacity, use of respirators, and motor scores for paralysis were evaluated. Of 182 patients included in this study, 33 (18%) developed pneumonia. Multiple logistic regression analysis revealed that low nutritional status, severe paralysis, and low vital capacity were significant risk factors for pneumonia. The severity of paralysis, respiratory dysfunction, and poor nutritional status can affect the occurrence of pneumonia after CSCI. In addition to respiratory management, nutritional assessment and intervention may play key roles in preventing pneumonia associated with spinal cord injury-induced immune depression. Nutritional care should be provided as soon as possible when the nutritional status of a patient worsens after an injury.

## Introduction

Respiratory diseases are the leading cause of death after spinal cord injury (SCI). Moreover, 65.1% of the deaths have been attributed to pneumonia^1^. The reported incidence of pneumonia varies widely (11–84%) owing to differences in the settings and diagnostic criteria used to diagnose pneumonia^2,3^. The treatment of pneumonia following SCI is challenging because of reduced respiratory function and atelectasis^4^. Because pneumonia is a common and serious complication, particularly in the acute phase^2,5^, elucidating the causes and mechanisms that reduce morbidity and mortality as well as the length of hospital stay and medical costs is crucial.

Previous studies have shown that the occurrence of pneumonia in SCI is associated with several factors, such as the severity of paralysis, severity of dysphagia^5^, male sex, presence of chest trauma, delayed intubation^2^, increased tracheal secretion^6^, enteral feeding, and mechanical ventilation^4^. In contrast, nutrition- and inflammation-related indicators have been used to predict surgical risks and postoperative complications^4,7,8^. Recent reports have shown a relationship between nutritional status and infectious complications in patients with gastric cancer^9,10^ or rheumatoid arthritis^11^. However, no previous studies have reported the effects of nutritional status on the incidence of pneumonia after cervical SCI (CSCI). We hypothesized that nutritional factors may affect the occurrence of pneumonia. This study aimed to elucidate the effects of nutritional factors on pneumonia after CSCI.

## Methods

### Participants

This study included patients with acute traumatic CSCI who were admitted within 3 days after injury and were followed up for at least 3 months. We retrospectively reviewed our database system^12^ and extracted data from October 2015 to June 2022. The exclusion criteria were as follows: no paresis, degenerative spinal disease, change in hospital owing to deteriorating general condition, transfer to a local hospital near home for rehabilitation, and other diseases causing dysphagia (e.g., brain injury, cerebral infarction, dementia, psychotic disorder, and neuromuscular disease).

### Evaluation

We diagnosed pneumonia as clinically defined pneumonia according to the Centre for Disease Control criteria^13,14^, which was used with the diagnostic algorithm for clinically defined pneumonia comprising combinations of the following timely available clinical and laboratory parameters: (1) at least one of the following: leukopenia or leukocytosis, altered mental status in patients aged ≥ 70 years, fever > 38 °C, (2) at least two of the following: new onset or changes in purulent sputum or respiratory secretions; new onset of cough/dyspnea; pathological auscultatory findings; worsening gas exchange such as O_2_ desaturations, increased O_2_ requirements, increased ventilator demand, or tachypnea, and (3) ≥ 2 serial chest radiographs with at least one of the following: new or progressive infiltrations and consolations or cavitation on a chest radiograph. In patients without an underlying pulmonary or cardiac disease, one definitive chest radiograph was acceptable^13,14^.

Nutritional status was assessed using both the prognostic nutritional index (PNI)^8^ and controlling nutritional status (CONUT) scores^15^, which objectively indicate nutritional and immunological conditions based on blood tests within 3 days after injury. The PNIs were calculated using the following formula: PNI = 10 × serum albumin (g/dL) + 0.005 × total lymphocyte count (/µL)^8,16^. The CONUT score was estimated using serum albumin concentration, peripheral lymphocyte count, and total cholesterol concentration^16^.

Two weeks after injury, dysphagia was evaluated using the dysphagia severity scale (DSS)^17^ and the functional oral intake scale (FOIS)^18^. The DSS evaluates aspiration or dysphagia using the following scores: (1) saliva aspiration; (2) food aspiration; (3) water aspiration; (4) occasional aspiration; (5) oral problems; (6) minimum problems; and (7) within normal limits^17^. The FOIS evaluates the functional intake of food or liquids using the following scores: (1) nothing by mouth; (2) tube-dependent with minimal attempts of food or liquid; (3) tube-dependent with consistent oral intake of food or liquid; (4) total oral diet of single consistency; (5) total oral diet with multiple consistencies but requiring special preparation or compensation; (6) total oral diet with multiple consistencies without special preparation but with specific food limitations; and (7) total oral diet with no restrictions^18^. We also used fiberoptic endoscopic evaluation of swallowing or videofluoroscopy to perform a detailed evaluation and determine the classification of DSS when patients were categorized as scale ≤ 4^5^.

Paralysis was evaluated using the American spinal injury association motor score^19^ 2 weeks after the injury. Regarding respiratory function, patient’s vital capacity was measured using a portable spirometer in the supine position 2 weeks after the injury.

### Statistical analysis

To identify the risk factors associated with the occurrence of pneumonia after CSCI, we analyzed the variables postulated to increase the risk of pneumonia^5,11^. The variables of patients with pneumonia were compared with those of patients without pneumonia using the student’s *t*-test and the Mann–Whitney U test. The chi-square test was used after the variables were categorized. A logistic regression model was used to compute the odds ratios (ORs), 95% confidence intervals (CIs), and cutoff values for increased risk of pneumonia. Receiver operator characteristic (ROC) curve analyses were performed to find the optimal cutoff values and area under the curve using univariate analyses. Age was categorized into two groups according to World Health Organization criteria: < 65 years and ≥ 65 years. PNI, motor score, and vital capacity were categorized into two groups according to the cutoff values dysphagia was categorized using the DSS based on whether aspiration occurred or not (≤ 4 and > 4). The logistic regression model was adjusted for age, sex, PNI, motor score, vital capacity, and DSS. All statistical analyses were performed using the JMP 17 software (SAS Institute Inc., Cary, NC, USA). Statistical significance was set at p < 0.05.

### Ethics approval and consent to participate

This study was approved by our institutional ethics committee (the Spinal Injuries Center Ethics Committee) and written informed consent was obtained from all participants prior to study participation. All methods were carried out in accordance with the Code of Ethics of the Declaration of Helsinki.

## Results

**Figure 1:**
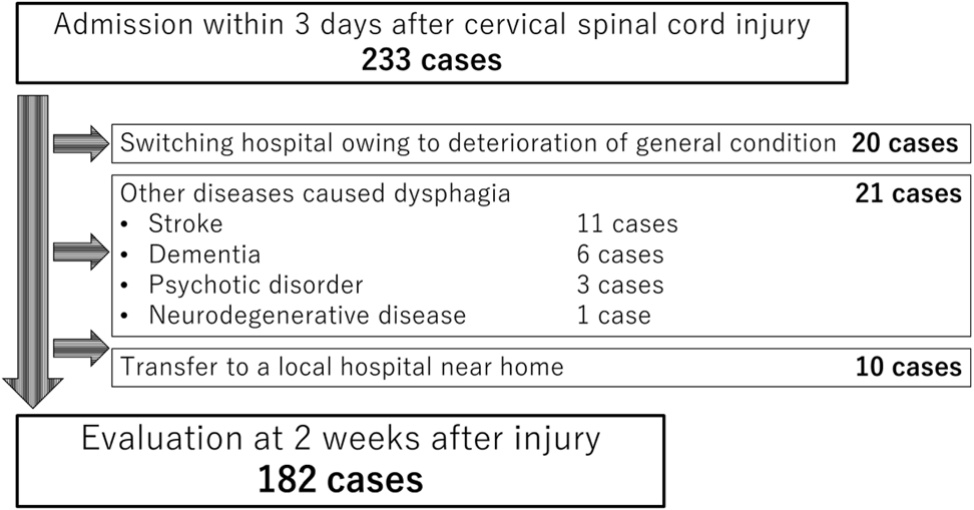
Flow chart of this study.

The flowchart of the study is presented in Fig. 1. A total of 233 individuals with acute traumatic CSCI were admitted to our center and included in this study. Of these, 51 individuals were excluded based on the exclusion criteria; 182 individuals met our inclusion criteria and were evaluated in this study.

The patient demographics are shown in Table 1. Of the 182 individuals, 33 developed pneumonia, indicating that the incidence of pneumonia was 18%. According to the univariate analysis, a high CONUT score and a low PNI, which indicate poor nutritional status, were significantly associated with the occurrence of pneumonia. Additionally, low DSS and low FOIS, which indicate severe dysphagia, were significantly associated with the occurrence of pneumonia. Low vital capacity, respiratory use, and low motor scores were also associated with the occurrence of pneumonia.

**Table 1:** Patient demographics.

	Total n = 182	Pneumonia		p-value
		( +)	( −)	
		n = 33	n = 149	
Age (years)	61.6 ± 16.5	65.2 ± 18.5	60.8 ± 16.0	n.s
Sex (male), n (%)	148 (81)	29 (88)	119 (82)	n.s
CONUT score	3 (4.5–1.5)	5 (6.5–3.5)	3 (2–4)	0.001
PNI	41.3 ± 5.2	38.4 ± 4.8	42.0 ± 5.1	< 0.001
Dysphagia severity scale	5 (6–4)	4 (3.5–4.5)	5 (4.5–5.5)	< 0.001
Functional oral intake scale	6 (7–5)	5 (4–6)	6 (5.5–6.5)	< 0.001
Vital capacity (mL)	1700 ± 882	1017 ± 462	1852 ± 882	< 0.001
Respirator, n (%)	11 (6)	8 (24)	3 (2)	< 0.001
Motor score	44.5 (11–78)	8 (0.5–15.5)	51 (17.5–84.5)	< 0.001

Values are given as number of patients (%), mean ± standard deviation, or median (interquartile range).

*CONUT* controlling nutritional status, *PNI* prognostic nutritional index, *n.s.* not significant.

Cutoff values were calculated using the univariate logistic regression analysis (Table 2). Four points on the CONUT score, 38.7 points on the PNI, scale four on the DSS, scale five on the FOIS, 1300 mL on vital capacity, and 17 points on the motor score were identified as the cutoff values for the occurrence of pneumonia. These cutoff values were used to categorize PNI, motor score, and vital capacity into two groups for logistic regression analyses: ≤ 38.7 and > 38.7, ≤ 17 and > 17, and ≤ 1300 mL and > 1300 mL, respectively. In addition, the area under the ROC curve of the PNI was higher than that of the CONUT score, indicating that the PNI was a better predictive parameter than the CONUT score.

**Table 2:** Cutoff value and area under the curve for pneumonia for each factor.

	Cutoff value	AUC
CONUT score	4	0.678
PNI	38.7	0.712
DSS	4	0.816
FOIS	5	0.823
Vital capacity	1300	0.806
Motor score	17	0.832

*CONUT* controlling nutritional status, *PNI* prognostic nutritional index, *DSS* dysphagia severity scale, *FOIS* functional oral intake scale, *AUC* area under the curve.

Details of individuals with and without pneumonia are shown in Table 3. Those with pneumonia showed a low PNI, low motor score, low vital capacity, and severe dysphagia based on the χ^2^ test. After adjusting for potential confounding factors, significantly elevated ORs were observed in those with PNI ≤ 38.7 (OR 3.35; 95% CI 1.19–9.39), motor score ≤ 17 (OR 7.13; 95% CI 2.46–20.66), and vital capacity ≤ 1300 mL (OR 5.06; 95% CI 1.69–15.1), indicating that low nutritional status, severe paralysis, and low vital capacity were significant risk factors for pneumonia.

**Table 3:** Risk factors for pneumonia in acute traumatic cervical spinal cord injury.

	Pneumonia		p-value^†^	Crude OR (95% CI)	Adjusted OR (95% CI)
	( +) n = 33	( −) n = 149			
Age (years), n (%)					
< 65	13 (39)	75 (50)		Reference	Reference
≥ 65	20 (61)	74 (50)	0.26	1.59 (0.72–3.36)	1.15 (0.39–3.47)
Sex, n (%)					
Male	29 (88)	119 (80)		Reference	Reference
Female	4 (12)	30 (20)	0.29	0.55 (0.15–1.53)	0.40 (0.10–1.65)
PNI, n (%)					
> 38.7	12 (36)	115 (77)		Reference	Reference
≤ 38.7	21(64)	34 (23)	< 0.001^†^	5.92 (2.69–13.60)*	3.35 (1.19–9.39)*
Motor score, n (%)					
> 17	8 (24)	120 (81)		Reference	Reference
≤ 17	25 (76)	29 (19)	< 0.001^†^	12.93 (5.50–33.43)*	7.13 (2.46–20.66)*
Vital capacity (mL), n (%)					
> 1300	7 (21)	105 (70)		Reference	Reference
≤ 1300	26 (79)	44 (30)	< 0.001^†^	8.86 (3.76–23.54)*	5.06 (1.69–15.1)*
Dysphagia severity scale, n (%)					
> 4	10 (30)	118 (79)		Reference	Reference
≤ 4	23 (70)	31 (21)	< 0.001^†^	8.75 (3.87–21.10)*	1.82 (0.56–5.89)

*PNI* prognostic nutritional index, *OR* odds ratio, *CI* confidence interval.

^†^p-value was calculated using the χ^2^ test.

*p < 0.05 using univariate or multivariate logistic analysis.

## Discussion

The importance of evaluating nutritional status to identify the risk of complications in digestive surgery has long been reported^8^. While previous studies have focused on the prognostic value of the PNI in patients with gastrointestinal cancer, only a few have identified a low PNI as a risk factor for infection after radical surgery for gastric cancer^9,10^. Regarding bone and joint disease in patients with rheumatoid arthritis, multivariate analyses showed that those with a low PNI (< 45) had a high incidence of infection^11^. The current study showed that a low PNI (≤ 38.7) was one of the significant risk factors for pneumonia after CSCI. To the best of our knowledge, this is the first study to demonstrate the relationship between nutritional status and pneumonia after CSCI. In addition, as the PNI had a higher area under the curve than the CONUT score when analyzing the cutoff value of patients with and without pneumonia, the PNI would be a better parameter than the CONUT score to predict this type of pneumonia.

Central nervous system injury induces secondary immunodeficiency, which is referred to as central nervous system injury-induced immunodepression^20^. Experimental SCI in rats induced early onset of immune suppression, which was referred to as SCI-immune depression syndrome, based on fluctuations in immune cell populations in the experimental rat model of SCI compared with sham-operated controls evaluated using fluorescence-activated cell sorting analysis^21^. In a mouse model of inducible pneumonia, high thoracic lesions that interrupted sympathetic innervation of major immune organs, but not low thoracic lesions, significantly increased the bacterial load in the lungs^22^. In this study, as a low PNI was statistically associated with the occurrence of pneumonia following CSCI, the PNI reflects not only the nutritional status but also the immune status.

Severe paralysis, which was indicated by a motor score of < 17 points in this study, was also a risk factor for pneumonia. Severe paralysis includes not only paralysis of the extremities but also paralysis of the diaphragm and intercostal and abdominal muscles, leading to an inefficient respiratory pattern. In cervical and high-thoracic SCI, there is also a loss of sympathetic innervation to the lungs, leading to unopposed parasympathetic activity and bronchospasm^23^. Our findings are consistent with those of previous reports indicating that the severity of paralysis increases the risk of pneumonia ^2,5,24^.

Low vital capacity, which was defined as a vital capacity of < 1300 mL in the current study, also affected the occurrence of pneumonia. Because a low % vital capacity, which correlates with a low motor score in CSCI^25^, is associated with weak cough and dysphagia^26^, respiratory dysfunction leads to difficulty in phlegm expulsion, resulting in atelectasis and pneumonia.

No previous study has identified cutoff values for respiration, paralysis, and nutritional status to predict the occurrence of pneumonia after CSCI. Nevertheless, this study had some limitations. Although we evaluated the earliest timing of blood sampling after injury to avoid the influence of pneumonia inflammation on the PNI, the severity of trauma may have affected the PNI value.

In conclusion, not only the severity of paralysis and respiratory dysfunction but also the poor nutritional status affected the occurrence of pneumonia after CSCI. In clinical practice, attention should be paid not only to respiratory status but also to nutritional factors in the acute phase after injury that would be associated with SCI-induced immune depression to avoid infection in individuals with CSCI. In addition to respiratory management, nutritional care should be provided as soon as possible when the nutritional status of a patient worsens after an injury. Moreover, early diagnosis and treatment of swallowing dysfunction associated with nutritional status^27^ after CSCI play a key role in improving nutritional status. Future study including the effect of the intervention for nutrition after CSCI is necessary to avoid complications.

## Conclusion

Not only the severity of paralysis and respiratory dysfunction but also the poor nutritional status affected the occurrence of pneumonia after CSCI. In addition to respiratory management, nutritional assessment and intervention may play key roles in preventing pneumonia.

## Data Availability

The datasets generated and/or analyzed in the current study are available from the corresponding author upon reasonable request.
